# Hospital-induced Delirium among Medical Older Adults: Evaluating the Veracity of Prognostic Models

**DOI:** 10.1093/geroni/igab046.3613

**Published:** 2021-12-17

**Authors:** Urszula Snigurska, Ragnhildur Bjarnadottir, Robert Lucero

**Affiliations:** 1 University of Florida College of Nursing, Gainesville, Florida, United States; 2 University of California Los Angeles, Los Angeles, California, United States

## Abstract

Several prognostic models have been developed and validated for delirium prediction among older adults. However, model development and validation studies need to be evaluated for risk of bias to establish the veracity of the prognostic models. This is a critical step before they can be implemented in clinical practice. Multiple systematic reviews have evaluated prognostic models of hospital-induced delirium. However, none of the existing systematic reviews evaluated the validity of models for non-surgical, medical hospitalized older adults. We conducted a scoping review to evaluate the validity of existing prognostic models of hospital-induced delirium in medical older adults. CINAHL, PsycINFO, PubMed, and Web of Science were searched for original studies. The database search yielded 4,312 records. Five studies were included in the qualitative synthesis. All the studies claimed to have developed valid prognostic models. However, the risk of bias assessment revealed that existing prognostic models of hospital-induced delirium in medical older adults are at a high risk of bias. Collectively, the statistical analysis was the greatest source of bias. Notably, while we have seen a proliferation of prognostic models for use in the surgical older adult population, efforts at developing prognostic models in the medical older adult population seem to have declined since the early 1990s. Newer methods of data collection, such as data mining of electronic health records, and statistical analysis, such as machine learning, have shown promise in accurate prediction of hospital-induced delirium while overcoming many challenges associated with manual data collection and traditional statistical analyses.

